# Development and application of a new biological nano-selenium fermentation broth based on *Bacillus subtilis* SE201412

**DOI:** 10.1038/s41598-023-29737-z

**Published:** 2023-02-13

**Authors:** Sisi Huang, Kan Yu, Liang Wen, Xiaoling Long, Jin Sun, Quxiao Liu, Zhuo Zheng, Wei Zheng, Hongmei Luo, Jinlong Liu

**Affiliations:** 1 Institute of Agricultural Economy and Technology, Hubei Academy of Agricultural Science, Wuhan, China; 2 Hubei Hualongxike Biotechnology Ltd., Huanggang, China

**Keywords:** Biotechnology, Plant sciences

## Abstract

In order to improve the functionality and additional value of agricultural products, this study developing nano-selenium fermentation broth and established a new application strategy of bio-nano-selenium by screening and identifying selenium-rich microorganisms. We isolated a new strain from tobacco waste and named it *Bacillus subtilis* SE201412 (GenBank accession no. OP854680), which could aerobically grow under the condition of 66,000 mg L^−1^ selenite concentration, and could convert 99.19% of selenite into biological nano-selenium (BioSeNPs) within 18 h. Using strain SE201412, we industrially produced the different concentrations of fermentation broth containing 5000–3000 mg L^−1^ pure selenium for commercial use. The synthesized selenium nanoparticles (SeNPs) were characterized by scanning electron microscopy (SEM), transmission electron microscopy (TEM), and nanoparticle tracking analysis (NTA). TEM and SEM results showed that SeNPs were distributed outside cells. NTA assay of fermentation broth indicated that the nanoparticles were spherical with an average particle size of 126 ± 0.5 nm. Toxicity test revealed that the median lethal dose (LD_50_) of the fermentation broth to mice was 2710 mg kg^−1^, indicating its low toxicity and high safety. In addition, we applied BioSeNP fermentation broth to rice and wheat through field experiments. The results showed that the application of fermentation broth significantly increased the total selenium content and organic selenium percentage in rice and wheat grains. Our findings provide valuable reference for the development of BioSeNPs with extensive application prospects.

## Introduction

Selenium is an essential trace element that plays an important role in the human health, especially in the fight against cancer^1^. Compared with inorganic and organic selenium, nano selenium with high particle dispersion, high bioactivity, low toxicity, and high human tolerability have attract extensive attention due to their broad application prospects^2^. Selenium nanoparticles (SeNPs) absorbed by the human body play a role in many physiological processes such as growth, reproduction, and immune regulation^3^, and they can protect the human body from the toxic effects of heavy metals and chemicals by reducing the production of reactive oxygen species (ROS)^4,5^. There are three main methods for the synthesis of SeNPs, namely, physical, chemical, and biological methods^6^. Compared with other methods, the biological method is more effective since the nano-selenium synthesized by biological method is more stable^7,8^. In biological synthesis process, biomolecules cover the surface of nano-selenium, thus blocking its aggregation and increasing its stability, and therefore biosynthesis method has become a new trend in the preparation of SeNPs^9^.

Currently, many plant extracts have been applied to preparing SeNPs. Song et al.^10^ prepared SeNPs with the particle size range of 50–150 nm through reaction of ascorbic acid (AA) and sodium selenite (Na_2_SeO_3_) using konjac glucomannan (KGM) as a stabilizer. Zhang et al.^11^ used *Lycium barbarum* polysaccharide (LBP) and *Lycium barbarum* protein conjugate (LBPP) respectively as stabilizer and dispersant to maintain the particle size of LBPP1-SeNPs within the range of 111.5–117 nm. Yilmaz et al.^12^ synthesized spherical SeNPs with the particle size range of 20–50 nm through the reaction of tarragon extract and sodium selenite. The results showed that the SeNPs synthesized from plant extracts were small in particle size, stable, non-cytotoxic, and environmentally friendly^13^.

Microbial green synthesis of selenium nanoparticles is currently a hot topic in the field of biosynthesis of selenium nanoparticles. Afzal et al.^14^ synthesized orange-red biogenic selenium nanoparticles (BioSeNPs) with a spherical shape and a particle size of 10.8 nm using cyanobacteria. El-Saadony et al.^15^ biosynthesized SeNPs with an average particle size of 56.91 ± 1.8 nm from lactic acid bacteria (LAB) isolated from human milk. Li et al.^16^ used the plant inter-rhizosphere-promoting bacterium *Rahnella aquatilis* HX2 to reduce selenite into BioSeNPs, and found that both FliC and OmpF proteins could control the assembly and stability of BioSeNPs. Sun et al.^17^ synthesized spherical SeNPs with a diameter of approximately 48 nm from selenium-rich strain 2322 *Rhodobacter erythropolis* through fermentation. Huang et al.^18^ isolated and identified a highly selenite-resistant (800 mM) strain *Providencia rettgeri* HF16 from coal mine soil, and based on this strain, they synthesized SeNPs with the particle size range of 120–295 nm. Tugarova et al.^19^ prepared SeNPs with the size range of 25–80 nm from strains Sp7 and Sp245 *Azospirillum brasilense*. Lian et al.^20^ prepared SeNPs with the particle size range of 70–90 nm from cell-free extracts of a novel yeast *Magnusiomyces ingens* LH-F1. Sara and Mohammad^21^ tested the ability of Enterococcus faecalis to transform sodium selenite into SeNPs, found that the transformed spherical BioSeNPs had a particle size ranging from 29 to 195 nm. All these studies prepare nano-selenium based on the transformation of selenium salts by microorganisms such as bacteria, fungi, and actinomyces. This microorganism synthesis method has mild reaction conditions, high stability, and environmental friendliness, and thus more suitable microbial species remain to be explored for the synthesis of SeNPs.

Although, there are many reports on the SeNP synthesis, most of them focus on the characterization and potential applications of SeNPs, and few reports on factory production and application of SeNPs are available. Therefore, in this study, a strain with high SeNP transformation efficiency and high sodium selenite tolerance was isolated from tobacco waste and characterized, and its factory-produced fermentation broth was subjected to toxicological tests and particle size analysis, and our developed nano-selenium fermentation broth was applied to rice and wheat, exhibiting high absorption, transport, and bioavailability.

## Materials and methods

### Strains and growth conditions

This strain was isolated from fermented tobacco waste from Enshi Tobacco Reroasting Plant in Hubei province in the early stage, named SE201412, and stored in China Typical Culture Preservation Center (Wuhan, China) with a storage number of CCTCC NO.ZM2015708. The 1 g tobacco sample was placed in a triangular bottle filled with 99 mL sterile water and shaken thoroughly. The solution was gradiently diluted 10^−2^–10^−8^ folds. The 0.1 mL diluted solution at different concentrations was plated evenly on LB medium. Each sample had 3 replicates. After being cultured at the constant temperature of 30 °C for 24 h, the 5 single colony strains with different morphologies were selected from the tobacco samples, and were transferred to the media on sterile plates for further culture. These five strains were cultured on LB medium added with different concentrations of sodium selenite (300–700 mg mL^−1^) for selenium resistance test. The strain with best growth state was selected, isolated, and purified using the streak plate method until no other bacteria appeared on the petri dishes.

LB solid medium was prepared by mixing 10 g L^−1^ sodium chloride, 10 g L^−1^ tryptone, 5 g L^−1^ yeast extract, 15 g L^−1^ agar powder and autoclaving at 121 °C for 20 min. Sodium selenite medium (500 mg L^−1^) was prepared by mixing 10 g L^−1^ sodium chloride, 10 g L^−1^ tryptone, 5 g L^−1^ yeast extract, 15 g L^−1^ agar powder and autoclaving at 121 °C for 20 min, followed by cooling to about 60 ℃ and the addition of 100 μL of prepared sodium selenite (500 mg mL^−1^) into every 100 mL LB solid medium to reach the final concentration of 500 mg L^−1^. Other concentrations of sodium selenite medium was prepared by adding corresponding volume of 500 mg mL^−1^ sodium selenite to LB solid medium.

### Characterization of strain

Bacterial gram staining experiment and cell morphology observations were performed under 100× microscope. Bacterial species were identified by the 16S rRNA method, and the sequences obtained were submitted to the NCBI database for BLAST alignment. The evolutionary tree was constructed using the Neighbour-Joining method using MAGA 7.0. The physiological and biochemical identification of bacteria was performed according to the Berger's manual.

### Localization of SeNPs in cells

To investigate the distribution characteristics of generated SeNPs in cells, bacterial cultures were analyzed by transmission electron microscopy and scanning electron microscopy. The bacteria in treatment groups were cultured in LB medium supplemented with 500 mg L^−1^ Na_2_SeO_3_, and those in the control group were cultured in LB medium with no Na_2_SeO_3_ supplementation.

### Production of BioSeNPs fermentation broth

**Figure 1:**
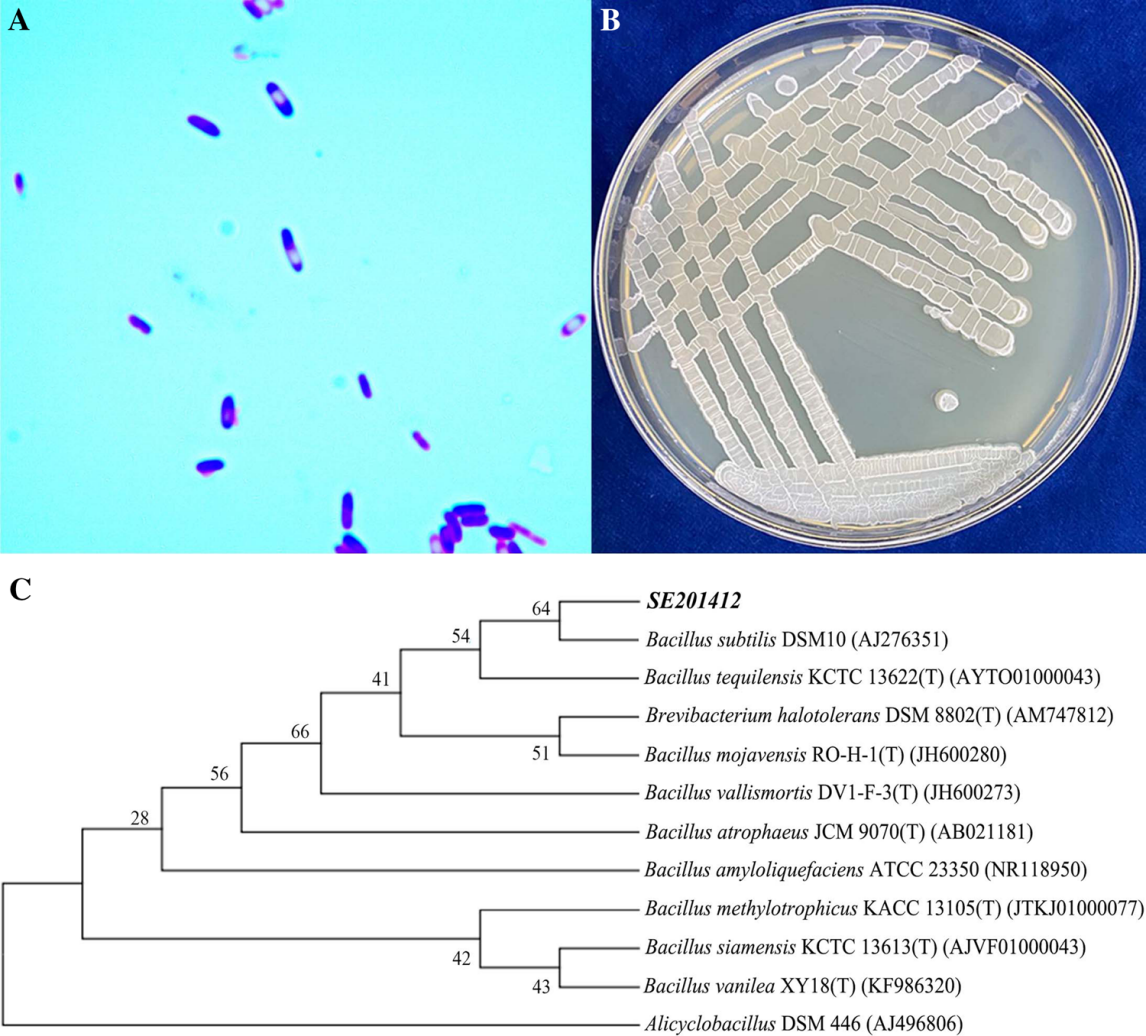
Characterization and identification of strain SE201412. (**A**) Morphology of strain SE201412 under optical microscope (×1000). (**B**) Culture of strain SE201412 in the medium without additional sodium selenite. (**C**) Phylogenetic tree of strain SE201412.

Purified strain SE201412 was selected from the sterile environment, cultured in the enlargement medium for 6–9 h at 30 °C and 150 r min^−1^, and transferred to the 200 L stainless steel self-controlled fermenter for fermentation (Fig. 1). LB medium was loaded into the fermenter to 70% of its volume and sterilized for 25 min under 0.12 MPa. When the medium temperature was lowered to 35 °C and bacterial growth reached the logarithmic growth phase (6 h), 10% sodium selenite was added, and at the end of the logarithmic growth phase, the remaining 90% sodium selenite was added. When sodium selenite was added, sterile air was introduced to keep the fermenter under positive pressure. The pressure and temperature of fermenter were set as 0.04–0.05 MPa and 30 ± 5 °C, respectively. In the fermenter, the pH value was 6.0, and the dissolved oxygen percentage was 60–100%. The speed of mixing was 190 r min^−1^. After fermentation for 18–24 h, 140 L fermentation broth was obtained.

### Conversion rate from inorganic selenium to organic selenium

The total and organic selenium in fermentation broth samples and mature grains of wheat and rice were determined by Enshi laboratory of Hubei Bureau of Geology and Mining. The selenium content was determined by atomic fluorescence spectrometry in ecogeochemical evaluation methods^22^. Oranic selenium conversion rate was calculated according to the following formula.

$$\mathrm{Organic \; Selenium \;Conversion\; Rate}=\frac{\mathrm{Organic \;Selenium \;Content}}{\mathrm{Total \;Selenium \;Content}}\times 100\mathrm{\%}$$

### Toxicity test of BioSeNPs fermentation broth

The toxicity test (GB 15193.3-2014) of BioSeNP fermentation broth was performed in a barrier environment animal room with animal experiment license number of SYXK (Zhe) 2016-0012 at room temperature 20.6–23.0 °C and relative humidity 57.9–64.2%. The test animals were ICR mice including 20 male and 20 female mice, provided by Zhejiang Experimental Animal Center with mouse production license number of SCXK (Zhe) 2014-0001. The BioSeNP fermentation broth sample was diluted with pure water and orally gavaged to mice.

The oral gavage doses were set as 10,000 mg kg^−1^, 4640 mg kg^−1^, 2150 mg kg^−1^ , and 1000 mg kg^−1^ by Horn's method, and the animals were fasted for 4 h before poisoning. The animal autopsy was performed at 14 days after poisoning.

### Characterization of particle size of SeNPs in fermentation broth

The particle size of SeNPs in fermentation broth prepared from strain SE201412 was measured by tracking Brownian motion trajectory using the NanoSight NS300 Nanoparticle Tracking Analyzer (NTA). The specific parameters of NTA assay were set as follows: NTA Version, NTA 3.4 Build 3.4.4; script used, SOP Standard Measurement 04-45-33PM 28O ~; camera type, sCMOS; laser type, Blue488; camera level, 9; slider shutter, 440; slider gain, 22; FPS, 25.0; number of frames, 1498; temperature, 23.9 ℃; viscosity, (Water) 0.912-0.912 cP; dilution factor, 1 × 10^−3^; syringe pump speed, 100; detection Threshold, 5; and max jump distance,17.0–20.0 pix (Auto).

### Application of BioSeNP fermentation broth to rice

We conducted a field plot trial by foliar spraying of 30 mg L^−1^ bioSeNP fermentation broth at different dosages (0 mL h^−2^, 3000 mL h^−2^, 4500 mL h^−2^, 6000 mL h^−2^, 7500 mL h^−2^, and 9000 mL h^−2^) in the early stage of filling of two rice varieties (E Xiang 2 and E Zhong 5), respectively. The total and organic selenium contents in the refined rice grains was measured after harvest. The field management was consistent with the local rice field management pattern.

### Application of BioSeNP fermentation broth to wheat

Twenty-four wheat varieties mainly promoted in the Yangtze River basin of China were selected for foliar spraying of 30 mg L^−1^ bioSeNP fermentation broth at the dose of 4500 mL hm^−2^ in the early filling stage. The experiments were performed in triplicates. After harvest, the total and organic selenium contents in the wheat grains were detected. The field management was consistent with the local wheat field management pattern.

### Statistical analysis

Non-parametric Kruskal–Wallis H test and Duncan’s multi-range test in SPSS version 22.0 were performed to assess differences of selenium content in rice and wheat, respectively. Origin version 9 was used for plotting. P < 0.05 was considered as statistically significant.

## Results and discussion

### Identification of strain SE201412

**Figure 2:**
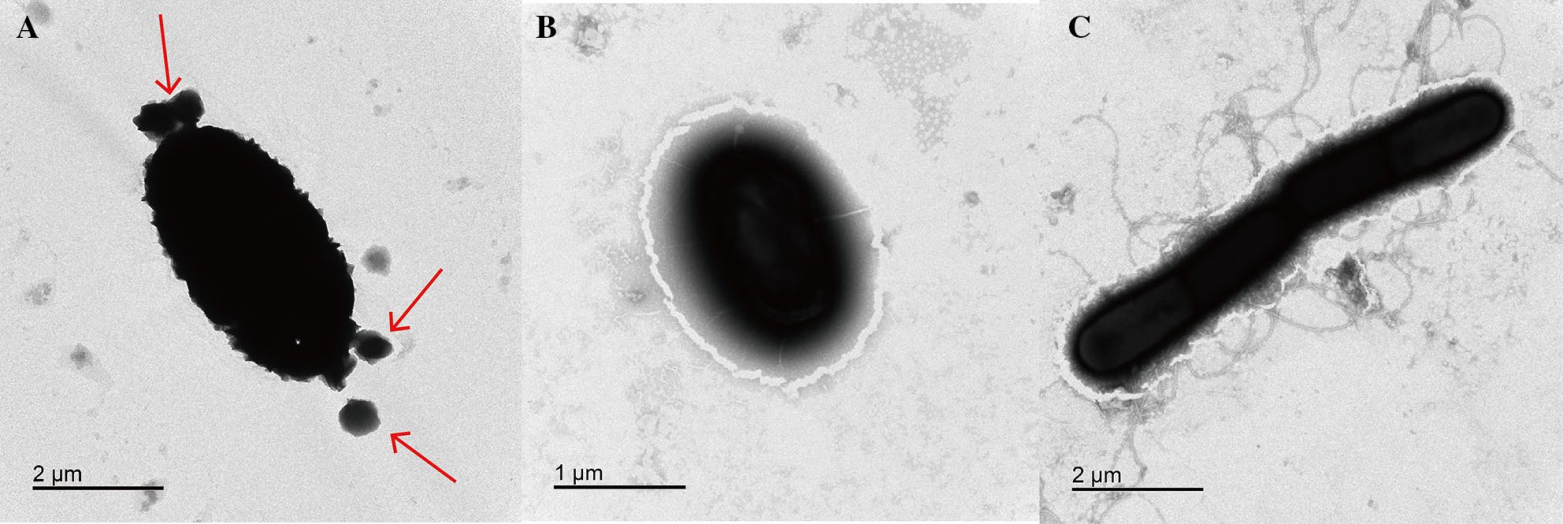
Transmission electron microscope (TEM) observation of strain SE201412. (**A**,**B**) Strain SE201412 after 6-h culture in 500 mg mL^−1^ sodium selenite medium. (**C**) Strain SE201412 after 6-h culture in sodium selenite- free medium.

We isolated a strain with high tolerance to sodium selenite and transformation efficiency from tobacco waste. The assays of morphological, physiological, and biochemical characteristics, electron microscope scanning, and 16S rRNA gene sequence analysis jointly indicated that this isolated strain was a new strain. This strain was named *Bacillus subtilis* SE201412, and its 16S rRNA sequence was submitted to the Genbank database with the registration number of OP854680. After gram staining, the color of SE201412 was observed to be purple (Fig. 1A), therefore this strain was positive. The colony was milk white and opaque with rough surface (Fig. 1B). The strain was stem-shaped with its endospore diameter of less than 1 µm (Fig. 2A). Moreover, bacterial body of this strain did not swell with lateral flagella, which conformed to the morphological characteristics of *Bacillus* (Fig. 2A). Phylogenetic and evolutionary analyses also showed that the SE201412 strain was closest to *Bacillus subtilis* DSM10 (AJ276351) (Fig. 1C). The 16S rRNA sequencing results indicated that the isolate strain SE201412 was similar to *Bacillus tequilensis* (AYTO01000043) and *Bacillus subtilis* subsp. *Inaquosorum* (AMXN01000021) with a 16S rRNA gene sequence similarity of 99.93% (Supplementary materials, Table S1). In addition, physiological and biochemical assays showed that strain SE201412 had the typical characteristics of *Bacillus subtilis* (Table 1). Therefore, isolate strain SE201412 was identified as *Bacillus subtilis* SE201412*,* and the 16S rRNA gene sequence obtained in this study was shown in Table S2, and the BLAST alignment results were shown in Table S1.

**Table 1:** Physiological and biochemical characteristics of bacterial strain SE201412.

Test item	Test result
Nitrate reduction into nitrite	+
Catalase	+
Hydrolyzed gelatin (protease)	+
Arabinose	+
Ribose	+
Fructose	+
Mannitol	+
Gram straining	+
Morphology of cell	Stem-formed
Flagellum	Lateral flagella
Mobility	Mobile
Alienation effect	Aerobic
Type of spore	Endospore

+ means positive.

The Bacillus sp. capable of preparing SeNPs have been reported as *Bacillus niabensis* OAB2^23^, *Bacillus paramycoides* SP3^24^, *Bacillus mycoides* SeITE01^25^, *Bacillus* sp. MSh-1^26^, *Bacillus amyloliquefaciens* SRB04^27^, etc. Bacillus subtilis is an aerobic bacterium widely distributed in soil and decaying organic matter, with fast growth rate, low nutritional requirements, efficient secretion of many proteins and metabolites, and no toxin production, which is a non-pathogenic and safe microorganism^28^. The strain SE201412 we identified for efficient preparation of selenium nanoparticles belonged to *Bacillus subtilis* of the genus Bacillus, which was not only corroborated that Bacillus has better tolerance and transformation effect on sodium selenite and is a good resource pool for screening efficient selenium-enriched bacteria, but also, illustrated that the strain SE201412 is a safe selenium-enriched microorganism.

### Characterization of SeNPs

**Figure 3:**
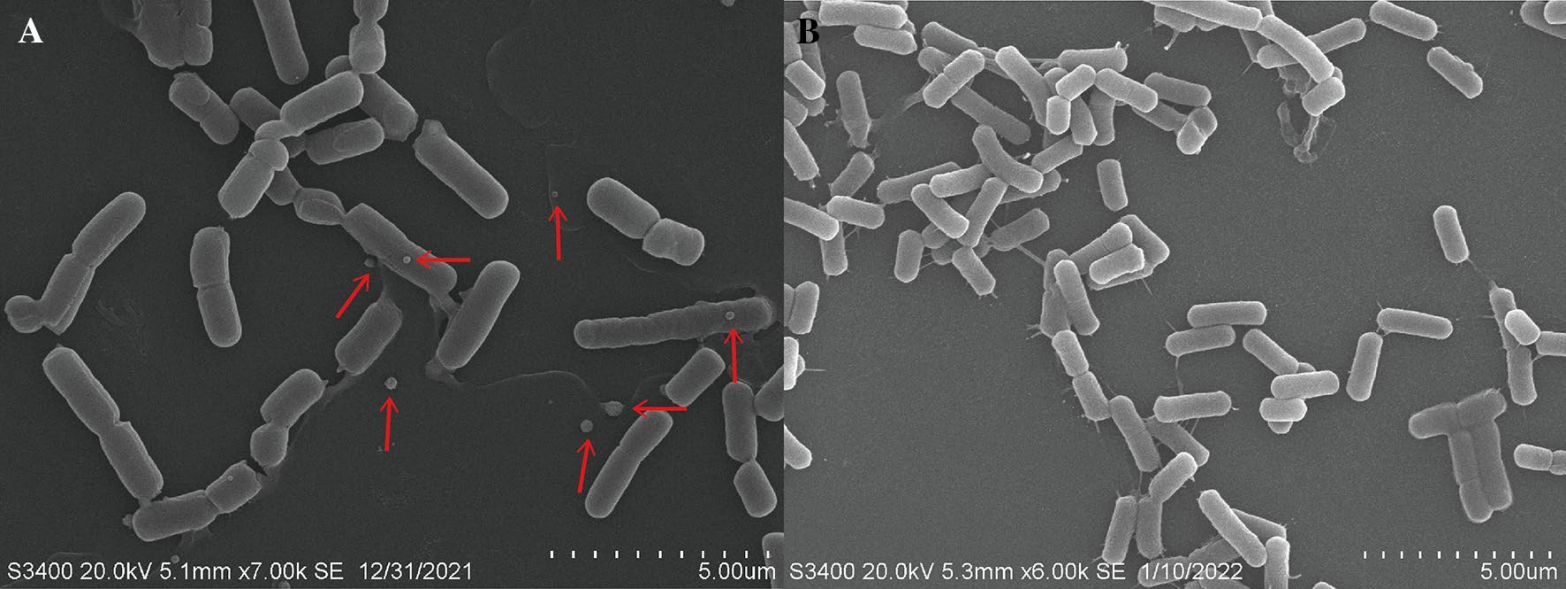
Scanning electron microscopy (SEM) observation of strain SE201412. (**A**) Strain SE201412 after 6-h culture in 500 mg mL^−1^ sodium selenite medium. (**B**) Strain SE201412 after 6-h culture in sodium selenite- free medium.

Since many bacteria have the ability to reduce sodium selenite to SeNPs, we investigated whether SE201412 strain could reduce selenite into Se^0^ to form SeNPs. TEM micrographs showed the presence of SeNPs produced by *Bacillus subtilis* SE201412 in bacterial cells and medium added with 500 mg L^−1^ sodium selenite (Fig. 2A), but no SeNPs were observed in SeNPs were not observed in the cells (Fig. 2B), while no SeNPs were observed in the medium added with no sodium selenite (Fig. 2C). After 6-h incubation in the medium containing 500 mg L^−1^ sodium selenite, SeNPs were distributed outside the cells. SEM micrographs clearly showed extracellular spherical SeNPs of different sizes (Fig. 3A), while no SeNPs were found in the selenium-free medium (Fig. 3B).

It was shown that thioredoxin reductase (TrxR) in Bacillus uses NADPH as an electron donor to directly reduce selenite to nanoselenium^29^. Compared to intracellularly distributed nanoselenium, the extracellular distribution of nanoselenium after synthesis results in nanoselenium being mobile and the synthesized nanoselenium being immediately present in the environment, and the extracellular nanoselenium is more easily recovered^28^. Therefore, the distribution of microbial-derived nanoselenium outside the cell is advantageous. *Bacillus subtilis* SE201412 could secrete nano-selenium with typical red color outside the cell after 6 h of culture, which had a shorter transformation time and better transformation effect compared with the existing reported selenium-enriched microorganisms^23–27^, but its specific transformation mechanism is not clear and needs further study.

### Particle size and toxicity of BioSeNP fermentation broth

**Figure 4:**
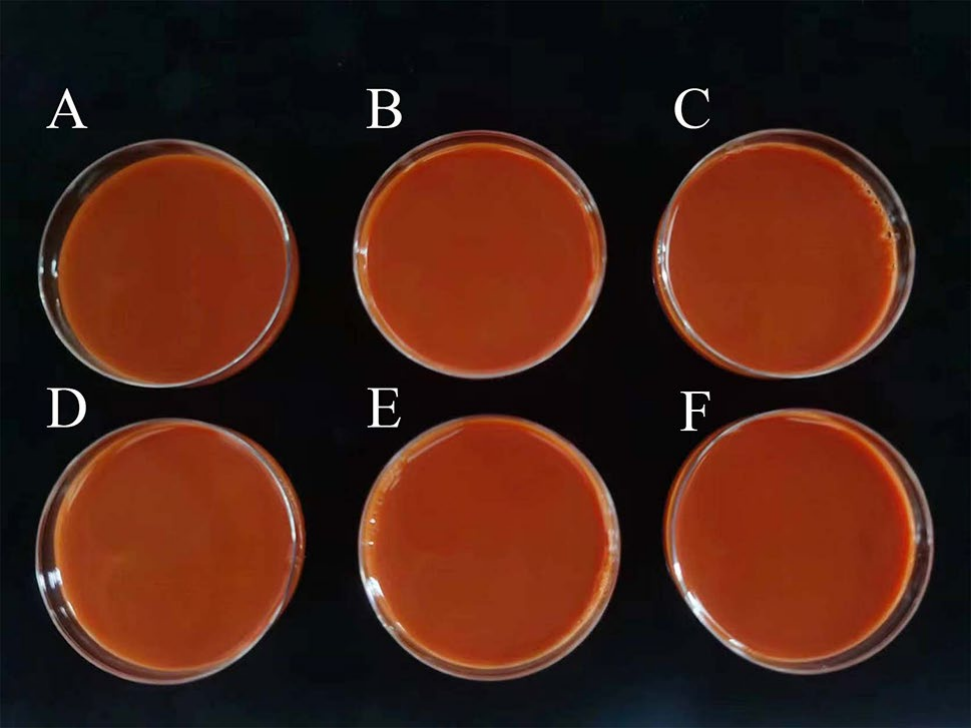
BioSeNP fermentation broth at different concentrations produced by strain SE201412. (**A**) Pure selenium content of 5,000 mg L^−1^. (**B**) Pure selenium content of 10,000 mg L^−1^. (**C**) Pure selenium content of 15,000 mg L^−1^. (**D**) Pure selenium content of 20,000 mg L^−1^. (**E**) Pure selenium content of 25,000 mg L^−1^. (**F**) Pure selenium content of 30,000 mg L^−1^.

Using strain SE201412, we produced the different concentrations of BioSeNP fermentation broth with pure selenium content of 5000 mg L^−1^, 10,000 mg L^−1^, 15,000 mg L^−1^, 20,000 mg L^−1^, 25,000 mg L^−1^ , and 30,000 mg L^−1^, respectively (Fig. 4), and the conversion rate from total selenium to organic selenium reached more than 99% (Table 2). The sodium selenite tolerance test found that the strain had high selenite tolerance and transformation ability, which could tolerate up to 66,000 mg L^−1^ (383 mM) sodium selenite and transform over 99% of sodium selenite into red SeNPs within 18 h. It has been reported that selenium tolerance of microorganisms generally ranges from 1 to 100 mM, and that of *Rhodopseudomonas palustris* N is 8 mM^30^. The selenium tolerance of *Streptomyces sp*. ES2-5 is 80 mM^31^, and the highest selenium tolerance of *Proteus mirabilis* YC801 is 100 mM^32^. However, the selenium tolerance capacity of the *Bacillus subtilis* SE201412 (383 mM) was much higher than that of these known bacteria. The SeNP synthesis capacity of different species of bacteria varies greatly since SeO_3_^2−^ and SeO_4_^2−^, as a toxic substance, affect the growth or metabolism of bacteria to various degrees. Therefore, bacteria need to tolerate certain concentration of SeO_3_^2−^ or SeO_4_^2−^ first, and then use their own detoxification mechanism to synthesize SeNPs^33^. Relevant studies have indicated that up to 40% selenium tolerance genes or proteins have been detected in *Bacillus*, which was considered to be an excellent selenium tolerance bacteria and an excellent bacterial resource for SeNP biosynthesis^34,35^.

**Table 2:** Selenium content in fermentation broth at different concentrations produced by strain SE201412.

Type^a^	Total selenium content (mg L^−1^)	Organic selenium content (mg L^−1^)	Percentage of organic selenium (%)
A	5017	4990	99.46
B	10,031	9962	99.31
C	14,989	14,914	99.50
D	20,004	19,898	99.47
E	25,053	24,960	99.63
F	30,068	29,824	99.19

^a^(A) A total of 11,000 mg sodium selenite was added in the average production of 1 L fermentation broth. (B) A total of 22,000 mg sodium selenite was added in the average production of 1 L fermentation broth. (C) A total of 33,000 mg sodium selenite was added in the average production of 1 L fermentation broth. (D) A total of 44,000 mg of sodium selenite was added for the production of 1 L fermentation broth. (E) A total of 55,000 mg of sodium selenite for the production of 1 L fermentation broth. (F) A total of 66,000 mg of sodium selenite for the production of 1 L fermentation broth.

**Figure 5:**
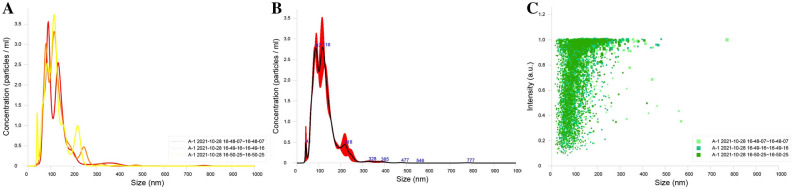
Particle size of bioSeNPs in fermentation broth. (**A**) Particle size distribution of three replicates. (B) Average particle size distribution of three replicates. Error bars indicate ± 1 standard error of the mean. (**C**) Density of particle size of bioSeNPs.

The particle size of SeNPs in the fermentation broth (10,000 mg L^−1^) produced by strain SE201412 was also examined (Fig. 5). The NTA results showed that the average particle size of SeNPs in the fermentation broth was 126 ± 0.5 nm, that D10 (when particle size distribution reaching 10%) was 78.6 ± 0.3 nm, that D50 (reaching 50%) was 113.4 ± 2.6 nm, and that D90 (reaching 90 50%) was 188.0 ± 7.8 nm. Compared with inorganic and organic selenium, SeNPs has higher bioactivity and lower toxicity. SeNPs synthesized by microbial reduction method is stable with excellent biological activity. Wang et al.^36^ successfully synthesized SeNPs with a particle size of 50–400 nm in vitro using *Bacillus subtilis*. Dhanjal and Cameotra identified a *Bacillus cereus* strain that can reduce selenite to SeNPs with a diameter of 150–200 nm under aerobic conditions^37^. Our NTA results showed that SeNPs generated by *Bacillus Subtilis* SE201412 had a stable structure and a particle size of 126 ± 0.5 nm. It is easy for SeNPs with a particle size less than 200 nm to enter cells and complete a series of metabolic activities, and thus they exhibit higher biological activity^38^.

We performed the toxicity tests of SeNP fermentation broth produced by strain SE201412 (Table 3). The results showed that all male and female mice died at 10,000 mg kg^−1^ and 4640 mg kg^−1^ doses. One male mouse died and 1 female animal died at 2150 mg kg^−1^ dose. No signs of poisoning or death were observed in males and females at the dose of 1000 mg kg^−1^ for 14 days. Gross autopsy results of all dead mice showed no pathological changes of poisoning. For male animals, LD_50_ (median lethal dose) was determined as 2710 mg kg^−1^, and for female animals, it was also 2710 mg kg^−1^. LD_50_ of chemically synthesized SeNPs and biosynthesized SeNPs has been reported to be 113.0 mg kg^−1^ and 198.1 mg kg^-^1, respectively, which was significantly lower than LD_50_ of selenate (1.6 mg kg^−1^), selenite (15.7 mg kg^−1^), and selenomethionine (27.0 mg kg^−1^)^39^. Our toxicity test of SeNPs fermentation broth produced by *Bacillus Subtilis* SE201412 showed an extremely low toxicity level with an LD_50_ value of 2710 mg kg^−1^, indicating that our prepared SeNPs fermentation broth was an extremely safe selenium supplementation resource with excellent application prospects.

**Table 3:** Acute oral toxicity test result.

Sex	Dose (mg kg^−1^)	Number	Body weight (${\overline{\text{X}}}$ SD) (g)				Death number	Mortality (%)
			Day 0	Day 7	Day 14	Body weight gain in 14 days		
Male	10,000	5	19.5 ± 1.18	–	–	–	5	100
	4640	5	21.2 ± 0.68	–	–	–	5	100
	2150	5	20.0 ± 1.06	24.6 ± 0.80	29.6 ± 0.97	9.9 ± 1.33	1	20
	1000	5	20.4 ± 1.21	24.9 ± 0.89	29.6 ± 1.06	9.2 ± 1.61	0	0
Female	10,000	5	20.4 ± 0.92	–	–	–	5	100
	4640	5	20.6 ± 1.05	–	–	–	5	100
	2150	5	19.9 ± 0.95	24.6 ± 0.48	28.1 ± 0.46	7.9 ± 0.95	1	20
	1000	5	19.2 ± 0.47	23.9 ± 0.65	27.8 ± 1.03	8.6 ± 0.77	0	0

### Application of bio-nano-selenium fermentation broth in agriculture production

SeNPs can be used as soil selenium supplement in selenium-deficient areas, and they can be used as exogenous selenium for the cultivation of selenium-rich crops, thus improving the growth performance and oxidation resistance of crops. Selenium is involved in the physiological metabolism of crops, and it plays an important role in improving yield and quality, and enhancing the stress resistance of crops^40^. Many studies have been carried out on the biological activity and biosynthesis of SeNPs^41^. At present, SeNPs have been applied in multiple crops such as corn^42^, tomato^43^, radish^44^ and shepherd's purse^45^. However, most of the studies of selenium enrichment strategies in rice and wheat are based on inorganic selenium (selenite and selenate), and there are few reports on nano-selenium.

**Figure 6:**
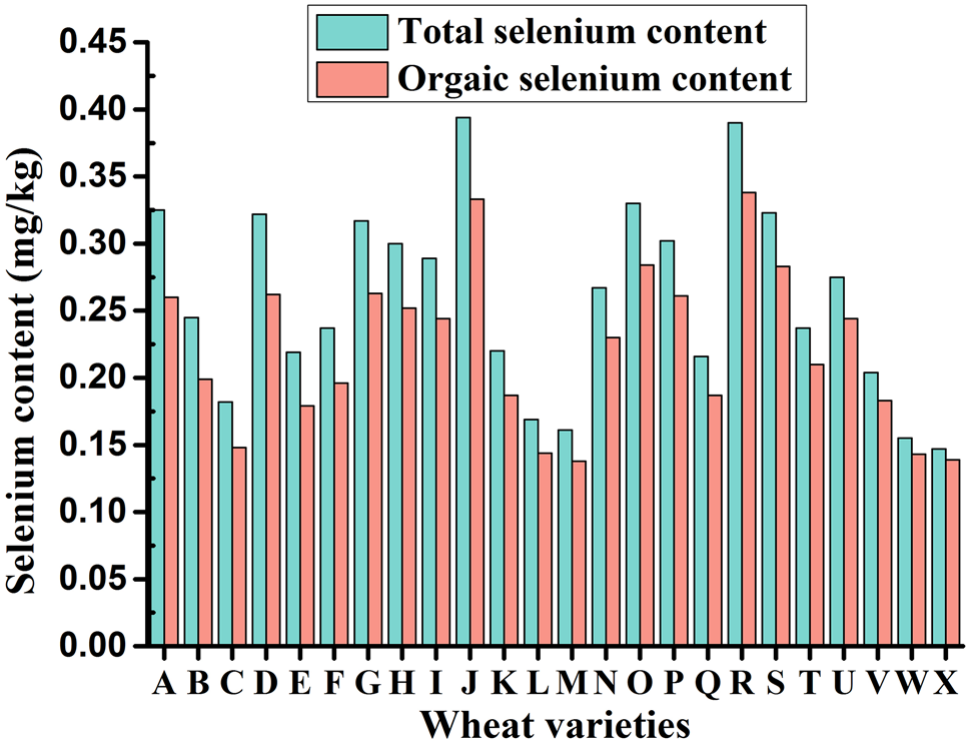
Total selenium content and organic selenium content in grains of 24 wheat varieties after foliar spraying of bioSeNP fermentation broth. (A) Xinmai 8811. (B) Ningmai 26. (C) Ermai 596. (D) Nannong 14Y106. (E) Wanximai 0638. (F) Ermai 251. (G) Eren 7. (H) Guohong 3. (I) Huamai 1168. (J) Huamai 2152. (K) Zhengmai 9023. (L) Ermai 170. (M) Xiangmai 35. (N) Xiangmai 55. (O) E Mai 23. (P) Hua mai 2668. (Q) Yang mai 20. (R) Xiang Mai 25. (S) E mai 18. (T) Yang mai 23. (U) E mai 580. (V) Hua Mai 2566. (W) E en 6. (X) E mai 352.

One previous study investigated the effect of selenite and selenate on the selenium content in grains of eight wheat varieties, and found the differences in selenium absorption of among different wheat varieties^46^. Another study conducted selenium biofortification by spraying selenate and selenite onto the wheat leaf surface, and found that both selenium sources significantly increased the selenium content in wheat grains, and after transformation, the proportion of organic selenium reached 72–93%^47^. In this study, BioSeNP fermentation broth at the same concentration and dosage was sprayed onto the leaves of 24 main wheat varieties in the middle and lower reaches of the Yangtze River in China. The results showed that there were differences in the absorption and utilization of the fermentation broth among 24 wheat varieties (Fig. 6), which was consistent with the previous reports^46^. However, one study has revealed that although there are differences in selenium absorption capacity among different wheat varieties, these differences were not statistically significant^48^. The possible reason might lie in that selenium absorption by wheat is also affected other factors such as environment temperature, soil selenium content, and application time of selenium supplement. In our study, the total selenium content in wheat grains ranged from 0.147–0.394 mg kg^−1^ with a mean value of 0.259 mg kg^−1^, and organic selenium content was 0.138–0.338 mg kg^−1^ with a mean value of 0.221 mg kg^−1^, accounting for 80–94.6% in total selenium with a mean percentage of 85.56%. Of 24 varieties, 19 wheat varieties exhibited a total selenium content greater than 0.2 mg kg^−1^, and their organic selenium percentage was above 80%. The percentage of organic selenium in wheat grains in our study was slightly higher that reported by Wang et al.^46^, which could be attributed to the smaller particle size of SeNPs in the fermentation broth, thus improving the absorption of organic selenium by wheat^47^.

**Figure 7:**
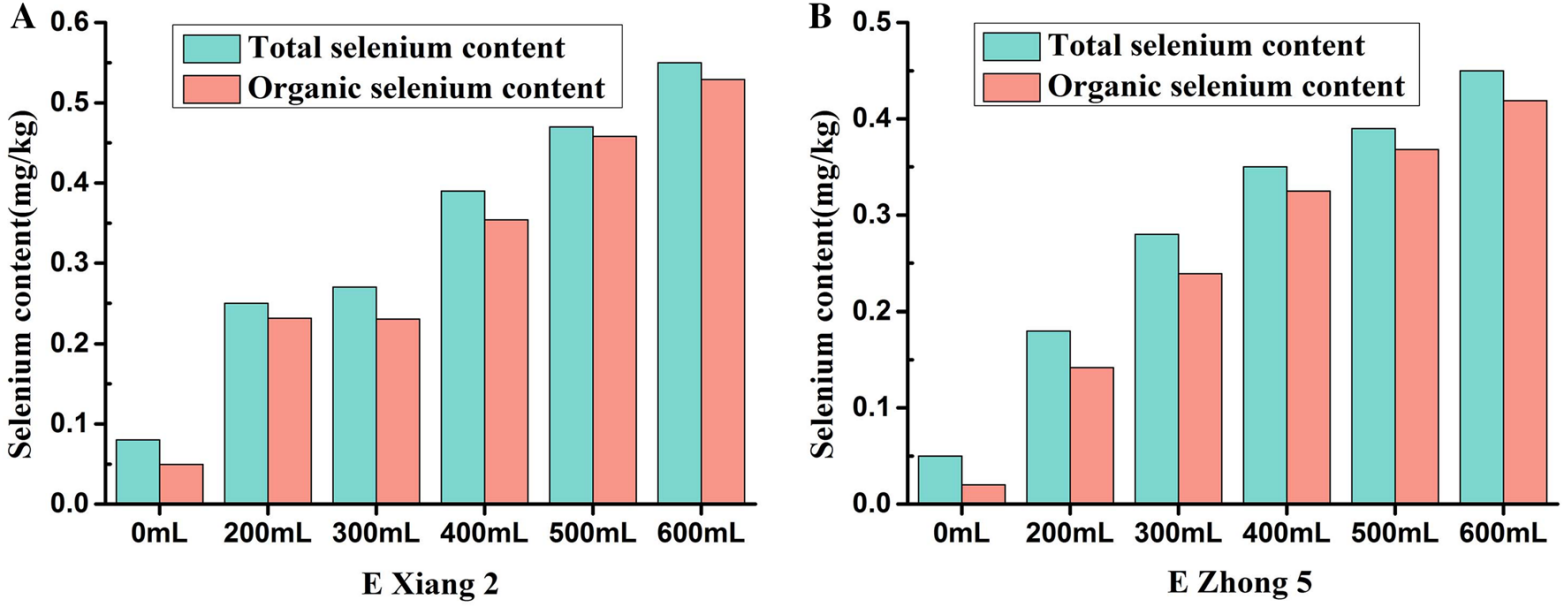
Total selenium content and organic selenium content in grains of two rice varieties under different selenium application doses. (**A**) Total selenium content and organic selenium content of E xiang 2. (**B**) Total selenium content and organic selenium content of E zhong 5.

Likewise, different rice varieties have different selenium absorption, transport, and accumulation abilities^49^. Therefore, it is also important to select rice varieties with strong selenium enrichment ability in the actual production of selenium-rich rice. We selected two rice varieties E Xiang 2 and E Zhong 5 and applied the same concentration of BioSeNP fermentation broth at different dosages by foliar spraying to them, and compared the total and organic selenium contents in refined rice grains of the two varieties (Fig. 7). Our data showed that the total and organic selenium contents in the refined rice grains of the two varieties in selenium treatment groups were higher than those in control group (without selenium application). The percentage of organic selenium in the selenium treatment group of E Zhong 2 reached above 85%, and the total selenium content increased by 212.5–587.5%, and the organic selenium content increased by 368.7–968.7%, compared with that of CK. The total selenium content increased with the increase of selenium application amount. After foliar selenium spraying, the total selenium content in selenium treatment group of E Zhong 5 increased by 560% with the mean value of 0.33 mg kg^−1^, and the organic selenium content ranged from 0.142–0.419 mg kg^−1^, accounting for 88.91%, which was 1393% higher than that in CK group. The total and organic selenium contents in refined rice grains were directly proportional to the spraying dosage. Our results were consistent with previous report that exogenous selenium supplementation significantly increased the selenium content in rice grains^50^. Since selenium is mostly accumulated in the brown rice layer, the selenium content in refined rice is far lower than that in brown rice^51^. Therefore, low selenium content in refined rice is difficult to meet the daily selenium requirement by people who consume refined rice in their daily diet. The total selenium content and organic selenium percentage in rice applied with BioSeNP fermented from *Bacillus subtilis* SE201412 were significantly increased. Our results also indicated that rice exhibited a high bioavailable efficiency of SeNPs in fermentation broth.

## Conclusion

This study investigated the ability of *Bacillus subtilis* SE201412 to convert sodium selenite into bio-nano-selenium. Our isolated and identified strain SE201412 could grow normally in the medium containing 66,000 mg L^−1^ (383 mM) sodium selenite, and its nano-selenium conversion rate exceeded 99% within 18 h. Additionally, fermentation broth with different selenium concentrations could be produced by this strain, and the average particle size of nano-selenium in fermentation broth was 126 nm. Toxicity test results showed that bioSeNP fermentation broth was safe with no side effects. The application of SeNP fermentation broth significantly increased the selenium content in wheat and rice grains and increased organic selenium percentage to above 80%. This study provides a new strategy for the application of bio-nano-selenium to different crops and varieties. Our developed bioSeNP fermentation broth based on *Bacillus subtilis* SE201412 exhibited great application prospects in agriculture. Our development and application of bioSeNP will contribute to meeting people's need for selenium supplementation.

## Supplementary Information

Supplementary Table S1.

Supplementary Table S2.

## Data Availability

The datasets used and/or analysed during the current study are available from the corresponding author on reasonable request.
