# Supplementary material for: Development and application of a new biological nano-selenium fermentation broth based on Bacillus subtilis SE201412
Source: Sci Rep. 2023 Feb 13;13:2560. doi: 10.1038/s41598-023-29737-z (PMC9925439; doi:10.1038/s41598-023-29737-z)
Supplement: Supplementary file 1 — Supplementary Table S1. [file 41598_2023_29737_MOESM1_ESM.docx]

**Table S1** BLAST comparison results of 16S rRNA gene sequencing for bacterial strain SE201412

| Rank | Species | Strain | Login Number | Genetic similarity/% | Difference/Total | Completeness/% |
| --- | --- | --- | --- | --- | --- | --- |
| 1 | *Bacillus tequilensis* | KCTC 13622(T) | AYTO01000043 | 99.93 | 1/1421 | 100 |
| 2 | *Bacillus subtilis* subsp. inaquosorum | KCTC 13429(T) | AMXN01000021 | 99.93 | 1/1421 | 100 |
| 3 | *Bacillus subtilis* subsp. *subtilis* | NCIB 3610(T) | ABQL01000001 | 99.86 | 2/1421 | 100 |
| 4 | *Brevibacteriumhalotolerans* | DSM 8802(T) | AM747812 | 99.79 | 3/1421 | 100 |
| 5 | *Bacillus subtilis* subsp. spizizenii | NRRL B-23049(T) | CP002905 | 99.79 | 3/1421 | 100 |
| 6 | *Bacillus mojavensis* | RO-H-1(T) | JH600280 | 99.72 | 4/1421 | 100 |
| 7 | *Bacillus methylotrophicus* | KACC 13105(T) | JTKJ01000077 | 99.65 | 5/1421 | 100 |
| 8 | *Bacillus vallismortis* | DV1-F-3(T) | JH600273 | 99.58 | 6/1421 | 100 |
| 9 | *Bacillus siamensis* | KCTC 13613(T) | AJVF01000043 | 99.58 | 6/1421 | 100 |
| 10 | *Bacillus amyloliquefaciens* subsp. *plantarum* | FZB42(T) | CP000560 | 99.51 | 7/1421 | 100 |
| 11 | *Bacillus atrophaeus* | JCM 9070(T) | AB021181 | 99.37 | 9/1421 | 100 |
| 12 | *Bacillus amyloliquefaciens* subsp. amyloliquefaciens | DSM 7(T) | FN597644 | 99.3 | 10/1421 | 100 |
| 13 | *Bacillus vanillea* | XY18(T) | KF986320 | 99.14 | 12/1390 | 95.72 |
| 14 | *Bacillus licheniformis* | ATCC 14580(T) | AE017333 | 98.24 | 25/1421 | 100 |
| 15 | *Bacillus sonorensis* | NBRC 101234(T) | AYTN01000016 | 98.03 | 28/1421 | 100 |
| 16 | *Bacillus aerius* | 24K(T) | AJ831843 | 98.03 | 28/1419 | 100 |
| 17 | *Bacillus altitudinis* | 41KF2b(T) | ASJC01000029 | 97.25 | 39/1420 | 100 |
| 18 | *Bacillus stratosphericus* | 41KF2a(T) | AJ831841 | 97.18 | 40/1420 | 100 |
| 19 | *Bacillus xiamenensis* | HYC-10(T) | AMSH01000114 | 97.18 | 40/1420 | 100 |
| 20 | *Bacillus aerophilus* | 28K(T) | AJ831844 | 97.18 | 40/1420 | 100 |
| 21 | *Bacillus safensis* | FO-36b(T) | ASJD01000027 | 97.04 | 42/1420 | 100 |
| 22 | *Bacillus pumilus* | ATCC 7061(T) | ABRX01000007 | 96.97 | 43/1420 | 100 |
| 23 | *Bacillus vietnamensis* | 15-1(T) | AB099708 | 96.02 | 54/1356 | 94.17 |
| 24 | *Bacillus aquimaris* | TF-12(T) | AF483625 | 95.92 | 58/1421 | 100 |
| 25 | *Bacillus oryzaecorticis* | R1(T) | KF548480 | 95.9 | 45/1098 | 75.63 |
| 26 | *Bacillus acidicola* | 105-2(T) | AF547209 | 95.85 | 59/1420 | 100 |
| 27 | *Bacillus haikouensis* | C-89(T) | KJ868191 | 95.78 | 60/1421 | 100 |
| 28 | *Bacillus shackletonii* | LMG 18435(T) | AJ250318 | 95.7 | 61/1419 | 100 |
| 29 | *Bacillus pakistanensis* | NCCP-168(T) | AB618147 | 95.58 | 62/1403 | 95.47 |
| 30 | *Bacillus galliciensis* | BFLP-1(T) | FM162181 | 95.57 | 63/1421 | 99.93 |
| 31 | *Bacillus marisflavi* | TF-11(T) | AF483624 | 95.36 | 66/1421 | 100 |
| 32 | *Bacillus seohaeanensis* | BH724(T) | AY667495 | 95.28 | 66/1398 | 95.12 |
| 33 | *Bacillus ginsengihumi* | Gsoil 114(T) | AB245378 | 95.21 | 68/1420 | 100 |
| 34 | *Bacillus gottheilii* | WCC 4585(T) | FN995266 | 95.2 | 68/1418 | 100 |
| 35 | *Bacillus dabaoshanensis* | GSS04(T) | KJ818278 | 95.14 | 69/1421 | 100 |
| 36 | *Bacillus mesophilum* | IITR-54(T) | JN210567 | 95.14 | 68/1399 | 97.18 |
| 37 | *Bacillus herbersteinensis* | D-1-5a(T) | AJ781029 | 95.13 | 69/1418 | 99.46 |
| 38 | *Bacillus sporothermodurans* | M215(T) | U49079 | 95.07 | 70/1421 | 99.46 |
